# Knockout crickets for the study of learning and memory: Dopamine receptor Dop1 mediates aversive but not appetitive reinforcement in crickets

**DOI:** 10.1038/srep15885

**Published:** 2015-11-02

**Authors:** Hiroko Awata, Takahito Watanabe, Yoshitaka Hamanaka, Taro Mito, Sumihare Noji, Makoto Mizunami

**Affiliations:** 1 Faculty of Science, Hokkaido University, Sapporo 060-0810, Japan; 2 Department of Biological Science and Technology, Faculty of Engineering, The University of Tokushima, Tokushima 770-8506, Japan.

## Abstract

Elucidation of reinforcement mechanisms in associative learning is an important subject in neuroscience. In mammals, dopamine neurons are thought to play critical roles in mediating both appetitive and aversive reinforcement. Our pharmacological studies suggested that octopamine and dopamine neurons mediate reward and punishment, respectively, in crickets, but recent studies in fruit-flies concluded that dopamine neurons mediates both reward and punishment, via the type 1 dopamine receptor Dop1. To resolve the discrepancy between studies in different insect species, we produced *Dop1* knockout crickets using the CRISPR/Cas9 system and found that they are defective in aversive learning with sodium chloride punishment but not appetitive learning with water or sucrose reward. The results suggest that dopamine and octopamine neurons mediate aversive and appetitive reinforcement, respectively, in crickets. We suggest unexpected diversity in neurotransmitters mediating appetitive reinforcement between crickets and fruit-flies, although the neurotransmitter mediating aversive reinforcement is conserved. This study demonstrates usefulness of the CRISPR/Cas9 system for producing knockout animals for the study of learning and memory.

Associative learning provides animals with the ability to adapt their behavior to the environment. Elucidation of basic reinforcing mechanisms for associative learning has been one of major subjects in neuroscience. We recently obtained evidence suggesting that the prediction error theory, which states that discrepancy, or error, between actual and predicted reward determines whether learning occurs^1^ and most successfully accounts for associative learning in mammals^2^, is applicable to associative learning in crickets^3^. The evidence we obtained thus suggests conservation of basic computational rules underlying associative learning between mammals and crickets. Thus, insects such as crickets now emerge as suitable animals to study basic neural mechanisms of associative learning.

In mammals, dopamine neurons in the midbrain are thought to play critical roles in mediating both appetitive and aversive reinforcement^2^. Roles of dopamine neurons in mediating appetitive reinforcement have also been documented in mollusks^4^. In crickets, we observed that administration of octopamine receptor antagonists (epinastine and mianserin) impair appetitive learning but not aversive learning, whereas administration of dopamine receptor antagonists (flupentixol, fluphenazine, chlorpromazine and spiperone) impairs aversive learning but not appetitive learning, thus suggesting that octopamine and dopamine neurons mediate appetitive and aversive reinforcement, respectively^5^,^6^,^7^,^8^. Octopamine and dopamine neurons have also been suggested to mediate appetitive learning (with sucrose reward) and aversive learning (with electric-shock punishment), respectively, in honey bees^9^,^10^. Yet, recent results obtained in the transgenic fruit-fly *Drosophila melanogaster* have yielded a different picture despite using sweet and electric-shock reinforcement as in the bee^11^,^12^,^13^,^14^,^15^. In the fly, different sets of dopaminergic neurons mediate both appetitive and aversive reinforcement via the type 1 dopamine receptor Dop1 (also referred to as DUMB, DopR1 or DopRI^16^). In this framework, octopamine neurons have only a peripheral role for sweet-taste sensing as their signals are relayed to dopamine neurons, which convey this information to central brain structures^11^,^12^,^13^,^14^,^15^. This critical difference between flies on the one hand, and crickets and bees on the other hand, is, therefore, that dopamine neurons mediate appetitive reinforcement signals in the former but not in the latter. We propose three possible reasons for this discrepancy, which we will study in the cricket. The first is that the difference is due to different methods used to knock-down dopaminergic signaling: while transgenesis provides a sophisticated way to silence neurotransmitter signaling in the fly, specificities of pharmacological antagonists used in the cricket are not perfect^17^,^18^. The second is different kinds of reward used. We used water reward in our previous studies on crickets, whereas most studies on fruit-flies used sucrose reward, except for one study that used water reward^19^. We thus paid attention to the possibility that dopamine conveys water reward but not sucrose reward in crickets. Thirdly, reinforcing mechanisms may not be uniform among insects. The third possibility is unusual, since it is generally believed that basic mechanisms of learning and memory are conserved among different insect species.

In order to resolve the issue disucssed above, we used the clustered regularly interspaced short palindromic repeats (CRISPR)/CRISPR-associated protein 9 (Cas9) system to produce knockout crickets for the *Dop1* gene. The CRISPR/Cas9 system was identified as a bacterial immune system, and RNA-guided DNA endonuclease, Cas9 protein, binds to short RNA fragments derived from the invading virus that are used as “guides” to target the destruction of viral DNA^20^,^21^. The sequence of the guide RNA defines the specificity of the Cas9 nuclease in a highly predictable manner, based on base pairing with the target DNA. Reprogrammed Cas9 protein with a desired guide RNA breaks targeted double-strand DNA, resulting in mutagenesis at the target sites^20^,^21^. The CRISPR/Cas9 system has been used to generate genome-edited organisms in a number of species^22^,^23^,^24^,^25^,^26^,^27^.

In this study, we used the CRISPR/Cas9 system to generate *Dop1* knockout crickets and tested their capability for appetitive and aversive olfactory learning. In crickets^16^, as in fruit-flies^11^ and honey bees^17^, a high level of *Dop1* gene expression is found in Kenyon cells of the mushroom body, which are known to play critical roles in olfactory learning in insects^28^,^29^,^30^,^31^. We show that *Dop1* knockout crickets are defective in aversive learning with sodium chloride punishment but not appetitive learning with water or sucrose reward. This study demonstrates the utility of the CRISPR/Cas9 system for producing knockout animals for the study of learning and memory.

## Results

### Generation of Dop1 knockout crickets using the CRISPR/Cas9 system

**Figure 1:**
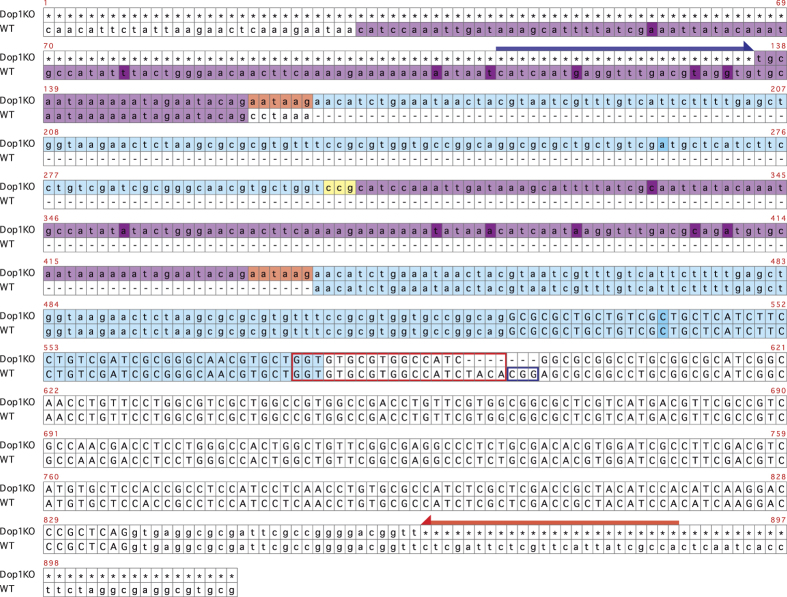
Target region of *Dop1* for the CRISPR/Cas9 system. The *Dop1* genomic sequence from *Dop1* knockout (KO) crickets (the upper rows) were aligned with the corresponding region of wild-type (WT) crickets shown in the lower rows. The target region and the adjacent PAM sequence for CRISPR/Cas9 (shown by red or blue squares, respectively) are located on the second exon of *Dop1*. *Dop1* knockout has a deletion of seven nucleotides over the target and the PAM in addition to two duplicated regions (purple- or light blue-shaded) and three insertions (red- or yellow-shaded). The same color-shaded regions show almost the same sequences except eight different nucleotides (dark-colored ones). The arrows show primers used to screen *Dop1* knockout crickets. The uppercase and lowercase letters show the coding sequence and the intron of *Dop1*, respectively. The dashes and asterisks indicate missing and unidentified regions, respectively.

For knocking out the *Dop1* gene by the CRISPR/Cas9 system, we designed a guide RNA, which was targeted to the second exon of the *Dop1* gene in the cricket *Gryllus bimaculatus* (see Methods; Fig. 1). *In vitro*-transcribed Cas9 mRNA and the guide RNA were co-injected into the cricket eggs. From 15 eggs injected with the guide RNA and Cas9 mRNA, five crickets grew up to adults and they were crossed with adults of wild-type crickets to obtain F1 offspring. The results of genotyping of F1 crickets showed that one strain had a mutation in the *Dop1* target region (Fig. 1). The mutated *Dop1* sequence of the knockout crickets had a deletion of seven nucleotides across the PAM (Protospacer Adjacent Motif) sequence and the target sequence we designed, and also had replacements of seven nucleotides, insertions of three nucleotides and two duplicated regions adjacent to the target region (Fig. 1). The F1 crickets had a *Dop1* mutation heterogeneously. The heterogeneous F1s were crossed with each other, and then their offspring, F2s, were used for breeding of the knockout strain. We checked the transcribed sequence of *Dop1* of the knockout cricket by RT-PCR or 3’RACE using specific primers of the cricket *Dop1*. None of the amplicons were found from cDNA of the knockout cricket brain (Supplementary Fig. S1). We used F3 or F4 having homogeneous *Dop1* mutation in the following learning experiments.

**Figure 2:**
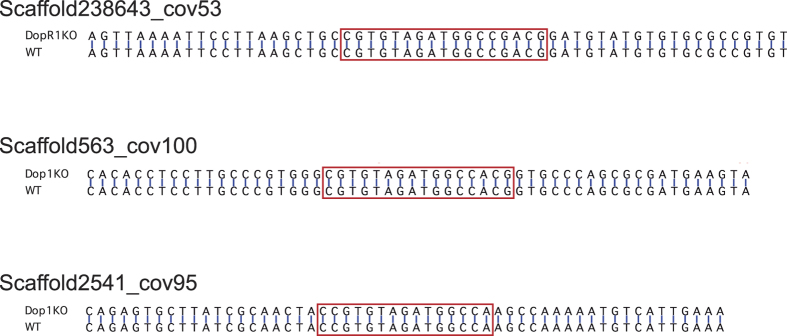
Potential regions for off-targeting effects in *Dop1* knockout crickets. The top three possible regions for off-target effects were sequenced. The potential regions of *Dop1* knockout (upper row) and wild type (lower row) were aligned. The potential regions are surrounded by red-colored squares. No differences between *Dop1* knockout and wild-type sequences were found in the potential regions or in their adjacent several dozen nucleotides regions.

The CRISPR/Cas9 system sometimes causes unexpected mutations in some regions having sequences similar to the original target because the system requires only 20 nucleotides adjacent to the PAM sequence (NGG)^20^,^21^. We thus sequenced possible off-target regions of the *Dop1* gene. A BLAST (Basic Local Alignment Search Tool) + survey of the genomic sequence of *Gryllus bimaculatus* (T.M. *et al.*, unpublished data) with the *Dop1* target sequence showed seven potential off-target regions, and we checked the sequences of the most similar three regions (Fig. 2). The three off-target regions did not have any mutations in *Dop1* knockout crickets compared to corresponding sequences in wild-type crickets. We concluded that the CRISPR/Cas9 system did not have an off-target effect in the *Dop1* knockout strain.

### *Dop1* knockout crickets exhibit defects in aversive learning but not in appetitive learning

**Figure 3:**
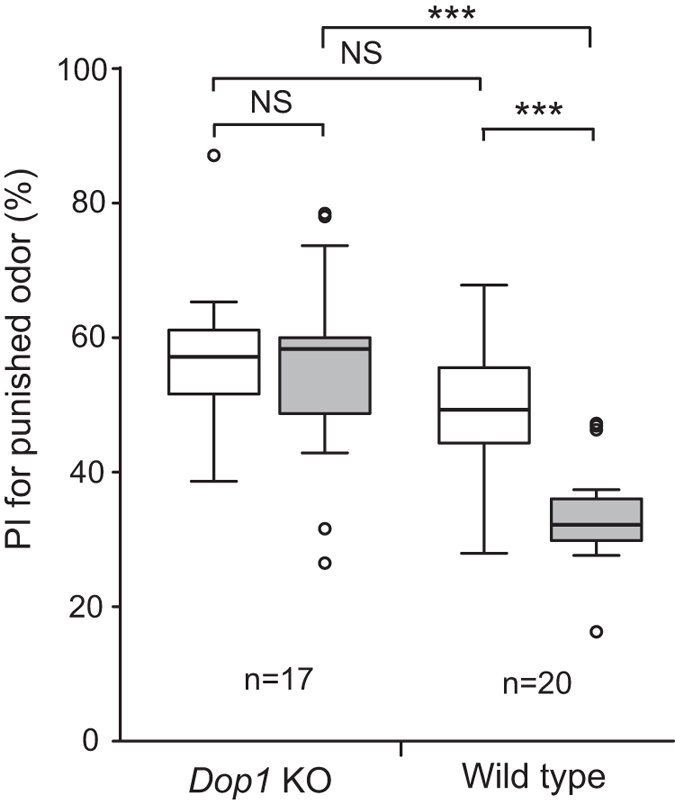
Impaired aversive learning in *Dop1* knockout crickets. *Dop1* knockout crickets and wild-type crickets were subjected to four-trial aversive conditioning with sodium chloride punishment, with an inter-trial interval (ITI) of 5 min. In both groups, relative preference between the punished odor (CS) and control odor was tested before and at 30 min after training. Maple odor and vanilla odor were used for conditioning, one of which was used as CS and the other was used as control. Preference indexes (PIs) for the punished odor before (white bars) and after (grey bars) training are shown as box and whisker diagrams. The line in the box is the median and the box represents the 25–75 percentiles. Whiskers extend to extreme values as long as they are within a range of 1.5× box length from the upper or lower quartiles. Any data outside the whiskers (outliers) are shown as dots. Odor preferences before and after training were compared by the WCX test. Odor preferences after training were compared between groups by the M-W test. The results of statistical comparisons are shown by asterisks (***P < 0.001, NS P > 0.05, adjusted by Holm’s method). The number of animals tested is shown at each data point.

*Dop1* knockout crickets exhibited normal viability and external morphology, and no obvious behavioral abnormality was detected by visual inspection. We first investigated whether *Dop1* knockout crickets exhibit aversive olfactory learning. *Dop1* knockout crickets and wild-type crickets (controls) were individually trained to associate an odor with 20% sodium chloride solution. The effect of training was evaluated by testing relative preference between aversively conditioned odor and control odor not used in training before and at 30 min after conditioning. Wild-type crickets exhibited a significant decrease of the preference for punished odor after training than that before training (Fig. 3: W = 202, p = 0.00019, Wilcoxon’s (WCX) test, adjusted by Holm’s method, sample numbers shown in legends), indicating that aversive learning is successful. In contrast, the preference for punished odor did not significantly differ before and after training in *Dop1* knockout crickets (Fig. 3: W = 72, p = 0.85, WCX test, adjusted by Holm’s method). Between-group comparison showed that the preference before training did not significantly differ between the two groups (Fig. 3. U = 227, p = 0.18, M-W test, adjusted by Holm’s method), but the preference for punished odor after training of *Dop1* knockout crickets was significantly greater than that of wild-type crickets (Fig. 3: U = 303, p = 0.00016; Mann-Whitney (M-W) test, adjusted by Holm’s method).

Because dopamine controls a variety of behaviors in insects^32^,^33^, we should be cautious that *Dop1* knockout crickets may be defective in sensory and motor functions necessary for learning and for responding to the odors in the test. All *Dop1* knockout crickets used in this study exhibited normal avoidance responses to NaCl solution: when an NaCl solution was attached to the mouth, they immediately retracted from the solution. Thus, defects in aversive learning were not due to impaired perception of NaCl punishment. Odor perception of *Dop1* knockout crickets was intact, as evidenced by intact appetitive odor learning described below. During testing, knockout crickets exhibited normal locomotory activity and exploration of odor sources. Moreover, knockout crickets that received appetitive conditioning training exhibited an increase in relative time to visit the conditioned odor compared to that before training (see below), indicating that sensory and locomotor functions necessary to perceive and visit the conditioned odor were intact in the knockout crickets.

**Figure 4:**
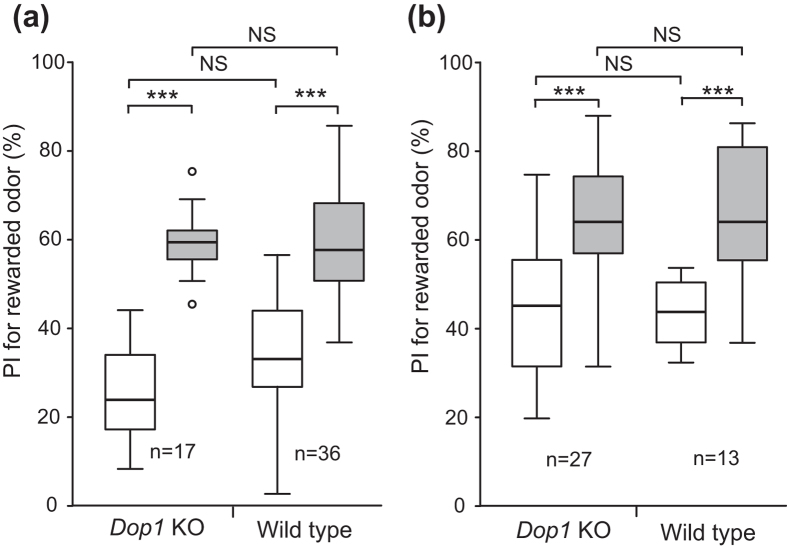
No impairment of appetitive learning with water or sucrose reward in *Dop1* knockout crickets. *Dop1* knockout crickets and wild-type crickets were subjected to two-trial appetitive conditioning with water (**a**) or sucrose (**b**) reward, with the ITI of 5 min. In both groups, relative preference between the rewarded odor (CS) and control odor was tested before and at 30 min after training. For conditioning with water reward, peppermint odor was used as CS and vanilla odor was used as control. For conditioning with sucrose reward, maple odor and vanilla odor were used, one of which was used as CS and the other was used as control. Preference indexes (PIs) for the rewarded odor before (white bars) and after (grey bars) training are shown as box and whisker diagrams. Odor preferences before and after training were compared by the WCX test. Odor preferences after training were compared between groups by the M-W test. The results of statistical comparisons are shown by asterisks (***P < 0.001, NS P > 0.05, adjusted by Holm’s method). The number of animals tested is shown at each data point.

We next studied whether *Dop1* knockout crickets acquire appetitive learning with water reward. *Dop1* knockout crickets and wild-type crickets ware individually trained to associate an odor with water reward. *Dop1* knockout crickets exhibited a significant increase of the preference for rewarded odor after training compared to that before training (Fig. 4a: W = 0, p = 0.00000000081, WCX test, adjusted by Holm’s method), as did wild-type crickets (Fig. 4a: W = 4, p = 0.00046, WCX test, adjusted by Holm’s method), indicating that the appetitive learning with water reward is successful. Between-group comparison showed that preferences for rewarded odor before and after training in *Dop1* knockout crickets did not significantly differ from those in wild-type crickets (Fig. 4a: before, U = 205.5, p = 0.11; after, U = 315, p  = 0.87; M-W test, adjusted by Holm’s method).

Finally, we studied whether *Dop1* knockout crickets exhibit appetitive learning with sucrose reward. Wild-type and *Dop1* knockout crickets were subjected to appetitive conditioning to associate an odor with 0.5 M sucrose solution. *Dop1* knockout crickets exhibited significantly increased preference for rewarded odor after training compared to that before training (Fig. 4b: W = 17, p = 0.000012, WCX test, adjusted by Holm’s method), as did wild-type crickets (Fig. 4b: W = 0, p = 0.00073, WCX test, adjusted by Holm’s method), indicating that the appetitive learning with sucrose reward is successful. Between-group comparison showed that preferences for rewarded odor before and after training in *Dop1* knockout crickets did not significantly differ from those in the wild-type crickets (Fig. 4b: before, U = 176 p = 1.0; after, U = 174.5, p = 0.99; M-W test, adjusted by Holm’s method). We thus conclude that the Dop1 dopamine receptor is required for aversive learning with sodium chloride punishment but is dispensable for appetitive learning with water or sucrose reward.

## Discussion

Biogenic amines play critical roles in associative learning in mammals^2^ and insects^15^,^29^,^34^,^35^. The aim of this study is to resolve the discrepancy between results of studies on crickets and fruit-flies concerning the roles of dopamine in mediating reinforcing signals in associative learning. We previously suggested that dopamine mediates aversive but not appetitive reinforcement in crickets^5^,^6^,^7^, whereas results of studies on fruit-flies led to the conclusion that dopamine and Dop1 dopamine receptor mediate both appetitive and aversive reinforcing signals^15^,^19^,^36^. In the present study, we generated *Dop1* knockout crickets using the CRISPR/Cas9 system and found that *Dop1* knockout crickets exhibit impairment in aversive learning but not in appetitive learning. This impairment was not due to impairment of sensory or motor functions necessary for learning and for responding to odors in the test. We thus conclude that Dop1 participates in aversive learning but not in appetitive learning in crickets. This differs from findings in fruit-flies that Dop1 is required for both appetitive and aversive learning^11^,^12^,^13^,^14^, suggesting that the neurotransmitter mediating appetitive reinforcement differs in crickets and fruit-flies, in contrast to conserved role of dopamine in mediating aversive reinforcement between them. It can be argued that dopamine receptors other than Dop1 may participate in appetitive learning in crickets. We suppose that this is less likely since we observed that all dopamine receptor antagonists we tested impaired aversive learning but not appetite learning in crickets^6^,^7^,^8^. However, more studies are clearly needed to resolve this issue.

The CRISPR/Cas9 system provides a convenient method for making modifications to a specific gene, but it should be cautioned that the system may have an off-target effect in that it causes mutations in sequences similar to the target gene^20^,^21^. DNA sequencing of some plausible off-target regions in *Dop1* knockout crickets, however, showed no mutations in areas with sequences similar to the target one. Thus, the impaired aversive learning is no doubt due to a defect of the *Dop1* gene. Since the CRISPR/Cas9 system enables easy simultaneous targeting of multiple genes^23^, it can be applied to investigate interactions between multiple molecules. It would be an intriguing question whether crickets with double knockout of *Dop1* and the gene coding for the octopamine receptor mediating appetitive reinforcement can still learn.

It can be argued that impaired aversive learning in *Dop1* knockout crickets may be due to defects in the formation of proper neural circuits in the brain during development, not due to the requirement of *Dop1* in aversive learning. In addition, since this study used only one allele of the *Dop1* mutant, the possibility that impaired aversive learning is due to other causes, such as secondary mutation and genetic background is not fully ruled out. However, the perfect match of the defects observed in *Dop1* knockout crickets and those caused by administration of dopamine receptor antagonists^5^,^6^,^7^ strongly argues for an acute role of *Dop1* in aversive learning. An approach to fully resolve this issue is to use RNAi, which is highly successful in crickets^16^,^37^,^38^, and we plan to perform a *Dop1* RNAi study. Moreover, results of further studies with RNAi, as well as studies on knockout crickets using CRISPR/Cas9 (this study) and TALENs^39^,^40^, should clarify the roles of dopamine receptors other than *Dop1*, as well as octopamine receptors, in appetitive and aversive learning in crickets.

We recently obtained evidence suggesting that the prediction error theory, which states that the discrepancy, or error, between actual and predicted reward determines whether learning occurs^1^ and is known to best account for associative learning in mammals^2^, is applicable to crickets^3^. Concerning the neurotransmitters mediating reinforcement in associative learning, we obtained evidence to suggest that octopamine mediates appetitive prediction error signals in crickets^3^, although whether dopamine mediates aversive prediction error signals remains to be investigated. In mammals, there is strong evidence that dopamine neurons in the midbrain mediate appetitive prediction error signals^2^ but whether they also mediate aversive prediction error signals remains controversial^41^,^42^. Future electrophysiological studies on dopamine and octopamine neurons projecting to the mushroom body, a higher-order associative center participating in olfactory learning^29^,^31^, will be promising to clarify the extent to which the mechanisms of prediction error computation are conserved among different phyla.

If neurotransmitters mediating appetitive reinforcement signals indeed differ between crickets and fruit-flies, it would be interesting to address the question of how such diversity has evolved. Since crickets (orthoptera) are evolutionary basal species and fruit-flies (diptera) are highly derived, and since octopamine is suggested to mediate appetitive reinforcement in honey bees^9^,^10^, one hypothesis that emerges is that the neurotransmitter mediating appetitive reinforcement altered from octopamine to dopamine at a point during the course of evolution of dipteran insects. To evaluate this and other possibilities, studies on species other than fruit-flies, crickets and honey bees, such as moths and cockroaches, are required. Moreover, since dopamine is thought to mediate appetitive and aversive reinforcement is mammals^2^ and to mediate appetitive reinforcement in mollusks^27^, consideration of evolutionary history of appetitive and aversive reinforcement signals among different phyla should become a fascinating future research subject. Importantly, the CRISPR/Cas9 system has enabled to edit targeted gene in essentially any organism and thus should greatly accelerate studies on neural mechanisms of associative learning in species in which sophisticated methods of genetic analysis have hitherto been not applicable.

## Methods

### Insects

Adult male crickets, *Gryllus bimaculatus*, at 1-2 weeks after the imaginal molt were used. They were reared in a 12 h:12 h light:dark cycle and were fed insect food pellets (Oriental Yeast, Tokyo, Japan) and water ad libitum. For three days prior to learning experiments with either NaCl punishment or water reward, crickets were given food *ad libitum* but were deprived of drinking water to enhance motivation to uptake water. For three days prior to learning experiments with sucrose reward, crickets were given water *ad libitum* but were deprived of food.

### Generation of *Dop1* knockout crickets

For generating a knockout line using the CRISPR/Cas9 system, we selected 20 bp of a target region for the plasmid of T7 promoter transcription (GGN_18_), which is followed by an NGG protospacer adjacent motif (PAM), using a web-based tool, ZiFiT Targeter Version 4.2 CRISPR/Cas nucleases (http://zifit.partners.org/ZiFiT/). The genomic sequence of *Dop1* of *G. bimaculatus*^16^,^43^ was surveyed and candidates of the target region were listed up. We checked nucleotide similarity between those candidate sequences and other biogenic amine receptors and chose a *Dop1*-specific sequence, which is located in the second exon of *Dop1*, as a target. For the guide RNA transcription, pDR274 vector^44^ (Addgene) was used. Two synthesized oligonucleotides including the target sequence (Fw; TAGGTGTGCGTGGCCATCTACA, Rv; AAACTGTAGATGGCCACGCACA) were annealed after phosphorylation of 5′ ends. The annealed oligonucleotides were inserted into BsaI digested and dephosphorylated pDR274 vector. DraI restriction enzyme was used to digest the target sequence-inserted pDR274 to prepare the transcription template. For the Cas9 mRNA transcription template, pMLM3613^44^ (Addgene) was linearized by PmeI restriction enzyme. Both the guide RNA and Cas9 mRNA were transcribed using an mMessage mMachine T7 Transcription Kit (Ambion) according to the manufacture’s instructions. Transcribed guide RNA and Cas9 mRNA (500 ng/μl each) were injected into cricket eggs within 5 hours after oviposition. The injection procedure for cricket eggs and the screening scheme for generating a mutant cricket line were previously described^39^,^40^. Because it was proved that the *Dop1* knockout crickets had an insertion of several hundred nucleotides on *Dop1* genomic DNA as shown in Fig. 1, genotyping PCR with KOD Fx Neo (TOYOBO) was performed using crude alkaline extracts from the tibia of the foreleg to distinguish Dop1-mutated offspring of injected crickets from wild-type ones. The primers used are shown as arrows in Fig. 1.

### Training and testing

We used classical conditioning and operant testing procedures described previously^5^,^45^. Either maple odor or vanilla odor was used as a conditioned stimulus (CS) in aversive conditioning^5^ and appetitive conditioning with sucrose reward. Peppermint odor was used as a CS in appetitive conditioning with water reward^46^. A syringe containing 20% NaCl solution, water or 0.5 M sucrose solution was used as an unconditioned stimulus (US). A filter paper soaked with the CS odor was attached to the needle of the syringe. A piece of filter paper was placed above the cricket’s head so as to present an odor, and then water, sucrose solution or NaCl solution was presented to the mouth. After the pairing trials, the air in the beaker was ventilated. The crickets were subjected to two trials of appetitive conditioning or four trials of aversive conditioning with an inter-trial interval (ITI) of 5 min. Crickets that did not exhibit aversive response to NaCl solution or appetitive response to water or sucrose solution were not used in the experiment. Such crickets were less than 3% in both wild-type and Dop1 knockout groups.

The procedure for the odor preference test was described elsewhere^5^,^45^. All crickets were subjected to odor preference tests before and at 30 min after conditioning. Crickets subjected to aversive conditioning trials with NaCl solution or appetitive conditioning trials with sucrose reward were subjected to dual-choice preference tests between maple odor and vanilla odor, one of which was used as a CS and the other used as a control stimulus, and crickets subjected to appetitive conditioning trials with water reward were subjected to preference tests between peppermint odor (CS) and vanilla odor (control odor). The floor of the test chamber of the test apparatus had two holes that connected the chamber with two odor sources. Each odor source consisted of a plastic container containing a filter paper soaked with odor essence, covered with a fine gauze net. Three containers were mounted on a rotative round holder and two of the three odor sources could be located simultaneously beneath the holes of the test chamber. Before the odor preference test, a cricket was transferred to the waiting chamber and left for about 4 min to become accustomed to the surroundings. Then the cricket was allowed to enter the test chamber and the test started. Two min later, the relative positions of the odor sources were exchanged by rotating the container holder. The preference test lasted for 4 min.

### Data analysis

We considered a cricket visited an odor source when the cricket probed the gauze net covering the odor source with its mouth or palpi. The time spent visiting each odor source was measured cumulatively. Relative preference for the conditioned odor was determined using the preference index (PI) defined as t_p_/(t_p_ + t_up_) × 100 (%), where t_p_ was the time spent exploring the odor paired with reward or punishment and t_up_ was the time spent exploring the odor not used in training of absolute appetitive conditioning or the odor presented alone without pairing with punishment in training of differential aversive conditioning. Because many of our data violated the assumption of a normal distribution, we used non-parametric tests. We compared odor preferences after training with those before training in each animal group by the Wilcoxon signed-rank test (WCX test). We also compared preferences after training between different groups by the Mann-Whitney U test (M-W test). For multiple comparisons, Holm’s method was used for adjusting the P value.

## Additional Information

**How to cite this article**: Awata, H. *et al.* Knockout crickets for the study of learning and memory: Dopamine receptor Dop1 mediates aversive but not appetitive reinforcement in crickets. *Sci. Rep.* **5**, 15885; doi: 10.1038/srep15885 (2015).

## Supplementary Material

Supplementary Fig S1
