# Supplementary material for: Knockout crickets for the study of learning and memory: Dopamine receptor Dop1 mediates aversive but not appetitive reinforcement in crickets
Source: Sci Rep. 2015 Nov 2;5:15885. doi: 10.1038/srep15885 (PMC4629116; doi:10.1038/srep15885)
Supplement: Supplementary Fig S1 [file srep15885-s1.pdf]

Title: Knockout crickets for the study of learning and memory: Dopamine receptor Dop1 mediates aversive but not appetitive reinforcement in crickets.

Authors: Hiroko Awata, Takahito Watanabe, Yoshitaka Hamanaka, Taro Mito, Sumihare Noji, Makoto Mizunami

Supplementary Figure S1

(a)

Dop1-1F	5′ -AACGACGAGGACGATCTGC-3’
Dop1-2F	5′ -ACGACTATCAGGTGAACGGGT-3’
Lock-Docking oligo dT primer	5′ -AAGCAGTGGTATCAACGCAGAGTACT$_{30}$VN-3′
Lock-Docking Anchor	5′ -AAGCAGTGGTATCAACGCAGAGT-3′

(b)

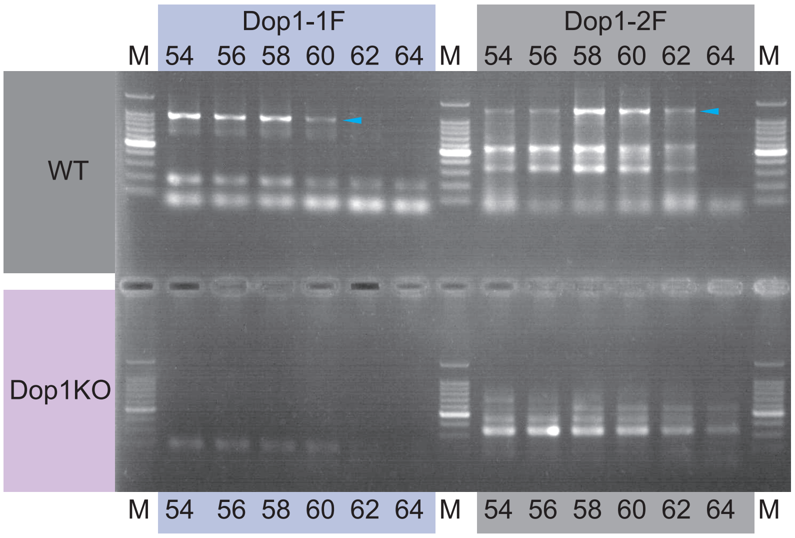

Title: Knockout crickets for the study of learning and memory: Dopamine receptor Dop1 mediates aversive but not appetitive reinforcement in crickets.

Authors: Hiroko Awata, Takahito Watanabe, Yoshitaka Hamanaka, Taro Mito, Sumihare Noji, Makoto Mizunami

Supplementary Figure Legends

Figure S1. Attempts to identify *Dop1* cDNA sequence of the *Dop1* knock-out (KO) by 3'RACE using *Dop1*-specific primers to determine whether mutated *Dop1* was expressed or not. (a) Primers used in the 3'RACE. The upper two primers were designed on the 1st exon of cricket *Dop1* gene. First, cDNA was created using SuperscriptIII Reverse Transcriptase (Life Technologies) Lock-Docking oligo dT primer and total RNA from cricket heads. The 3'RACE was then performed using the *Dop1*-specifid primer (Dop1-1F or Dop1-2F) and Lock-Docking Anchor according to the manufacture's instructions. (b) Electrophoresis photograph of the *Dop1* 3'RACE. Though we tried the amplification with some different conditions on the annealing step several times, any *Dop1*-specific fragments derived from the *Dop1*KO mutant were not amplified. The expected amplicons of *Dop1* were obtained using the wild-type (WT) cricket cDNA with the same conditions (arrowheads). We sequenced some amplicons shorter than we expected for *Dop1* amplicions, derived from the *Dop1*KO mutant (seen in the photograph), and confirmed that they were non-specific ones. M: 200 ng of 100 bp DNA ladder marker (TaKaRa). The numbers show the temperatures at the annealing step of the 3'RACE.
